# Barriers and Enablers to the Adoption of a Healthier Diet Using an App: Qualitative Interview Study With Patients With Type 2 Diabetes Mellitus

**DOI:** 10.2196/49097

**Published:** 2023-12-19

**Authors:** Jonas Montilva-Monsalve, Bruna Dimantas, Olga Perski, Leslie Morrison Gutman

**Affiliations:** 1 Centre for Behaviour Change, Department of Clinical, Educational and Health Psychology University College London London United Kingdom

**Keywords:** behavior change techniques, diabetes, apps, smartphone, enablers, barriers, mobile phone

## Abstract

**Background:**

Adopting a healthy diet is one of the cornerstones of type 2 diabetes (T2D) management. Apps are increasingly used in diabetes self-management, but most studies to date have focused on assessing their impact in terms of weight loss or glycemic control, with limited evidence on the behavioral factors that influence app use to change dietary habits.

**Objective:**

The main objectives of this study were to assess the enablers and barriers to adopting a healthier diet using the Gro Health app in 2 patient groups with T2D (patients with recently diagnosed and long-standing T2D) and to identify behavior change techniques (BCTs) to enhance enablers and overcome barriers.

**Methods:**

Two semistructured qualitative interview studies were conducted; the first study took place between June and July 2021, with a sample of 8 patients with recently diagnosed (<12 mo) T2D, whereas the second study was conducted between May and June 2022 and included 15 patients with long-standing (>18 mo) T2D. In both studies, topic guides were informed by the Capability, Opportunity, Motivation, and Behavior model and the Theoretical Domains Framework. Transcripts were analyzed using a combined deductive framework and inductive thematic analysis approach. The Behavior Change Wheel framework was applied to identify appropriate BCTs that could be used in future iterations of apps for patients with diabetes. Themes were compared between the patient groups.

**Results:**

This study identified similarities and differences between patient groups in terms of enablers and barriers to adopting a healthier diet using the app. The main enablers for recently diagnosed patients included the acquired knowledge about T2D diets and skills to implement these, whereas the main barriers were the difficulty in deciding which app features to use and limited cooking skills. By contrast, for patients with long-standing T2D, the main enablers included knowledge validation provided by the app, along with app elements to help self-regulate food intake; the main barriers were the limited interest paid to the content provided or limited skills engaging with apps in general. Both groups reported more enablers than barriers to performing the target behavior when using the app. Consequently, BCTs were selected to address key barriers in both groups, such as simplifying the information hierarchy in the app interface, including tutorials demonstrating how to use the app features, and redesigning the landing page of the app to guide users toward these tutorials.

**Conclusions:**

Patients with recently diagnosed and long-standing T2D encountered similar enablers but slightly different barriers when using an app to adopting a healthier diet. Consequently, the development of app-based approaches to adopt a healthier diet should account for these similarities and differences within patient segments to reduce barriers to performing the target behavior.

## Introduction

### Background

Type 2 diabetes (T2D) is a chronic noncommunicable disease, with an increasing prevalence in high-income countries such as the United Kingdom, where an estimated 4.2 million people live with the disease [1]. T2D care relies mainly on patient self-management, which encompasses behaviors such as monitoring blood sugar levels, engaging in physical activity, and adopting a healthy diet. With respect to adopting a healthy diet, tailoring the diet to individual patient needs is essential for T2D management, which implies taking into consideration the differences that exist between patients with recently diagnosed T2D (<1 y after diagnosis), who require an onboarding on dietary changes, and patients with long-standing T2D (>1 y after diagnosis), who may struggle to sustain dietary changes over time. In this context, apps have been used to support patients with T2D in adopting a healthy diet, demonstrating favorable effects in terms of improved glycemic control [2] and weight loss [3]. However, there is limited evidence on the behavioral enablers and barriers influencing user engagement with apps when adopting a healthy diet, as well as how these enablers and barriers may differ between patients with recently diagnosed T2D and those with long-standing T2D.

The Behavior Change Wheel (BCW) can be useful in this regard, offering a theoretically based, systematic framework to examine enablers and barriers to using a T2D app to adopt a healthy diet, which can then be linked to corresponding intervention strategies or behavior change techniques (BCTs) for optimization. Using BCW, we first highlighted the enablers and barriers to adopting a healthy diet before and when using an app in patients with recently diagnosed and long-standing T2D and then identified BCTs that specifically address the common and distinct barriers for both patient groups.

### Current T2D Management

Diabetes care is primarily dependent on patient self-management, which, if not performed, increases the risk of premature death, blindness, and kidney failure [4]. T2D self-management consists of the development of knowledge or awareness to survive the complexity of diabetes in a social context, including behaviors such as monitoring of blood sugar, being physically active, improving medication adherence, and adopting a healthy diet [5].

A healthy diet plays a central role in the management of T2D in patients with a recent and long-standing diagnosis, as it contributes to common goals such as achieving glycemic targets (ie, inducing a reduction in hemoglobin A_1c_) and weight loss in patients who are overweight and obese [6]. Although there is no single diet that is unanimously endorsed for patients with T2D, adapting and tailoring diets to their needs (especially the needs of patients with recently diagnosed T2D) is an essential first-line intervention for the management of T2D.

Multiple factors prevent patients with prediabetes and newly diagnosed T2D from changing their dietary patterns, whereas patients with a long-standing diagnosis struggle to maintain dietary changes over time. Pikkemaat et al [7] found several barriers to dieting among patients with recently diagnosed T2D, including a lack of knowledge about the correct diet to adopt, absence of self-regulatory skills (eg, low self-control and lack of self-monitoring skills), low motivation, low self-efficacy, and lack of social and medical support. In the case of patients with a long-standing diagnosis, an important factor that affects the sustainability of lifestyle behavioral changes is the time since diagnosis (ie, duration of time living with diagnosis); receiving a diagnosis from a health care professional may increase patients’ awareness and motivation for lifestyle changes, but this motivation may fade over time [8]. In addition, sustainable lifestyle changes must be adopted and endorsed by patients, coopted into their social setting (ie, endorsed by family and friends), and supported by health care professionals [9]. In view of these factors, it is paramount to have effective early behavior change interventions and target the underlying influences that prevent patients with recently and long-standing T2D from adopting or maintaining a healthier diet. Among these, digital technologies are increasingly being used to remotely support patients with T2D in their lifestyle management.

### Digital Behavior Change Interventions

Delivering theory- and evidence-based, cost-effective, highly available, flexible, and engaging real-life interventions has become a focus of T2D intervention development [10]. Consequently, digital behavior change interventions (eg, *apps*) have become increasingly popular and stand to bridge the gaps in health care outreach, particularly among underserved populations, who can be readily accessed via the web [11]. The use of apps has been demonstrated to improve glycemic outcomes in people with type 1 diabetes and T2D [2]. A meta-analysis also reported that apps for T2D management have a positive effect on weight loss, with 14 studies that enrolled 2100 patients showing apps that could significantly reduce body weight, particularly among patients who were obese [3]. To date, most studies have focused on assessing the impact of using an app for T2D management in terms of weight loss or glycemic control outcomes, but there is limited evidence on the behavioral enablers and barriers to change dietary habits using an app, which is an important element to understand when promoting effective behavior change.

Few studies to date have evaluated T2D apps from a behavioral perspective, and they have focused on identifying the BCTs that have been included in T2D apps. Hoppe et al [12] conducted a review of 10 diabetes apps, assessing the number of BCTs included, and found that the average number of BCTs was 4.4, out of a possible maximum of 26 BCTs, as proposed by a taxonomy of BCTs [13]; the most common BCTs were “self-monitoring of behavior,” “intention formation,” “goal setting” and “feedback on performance.” Consistent with these findings, Priesterroth et al [14] assessed 56 diabetes apps using a taxonomy of BCTs, which is modified from the taxonomy given by Michie et al [15], and found that an average of 7.4 BCTs were implemented in each app, including “self-monitoring of behavior,” “feedback on behavior,” as well as “self-monitoring of outcomes of behavior” among the most frequently used BCTs. Although these findings provide insights into the BCTs used in T2D apps, these studies have neither systematically identified which BCTs could best support self-management behaviors, such as adopting a healthy diet using an app, nor reported findings for individual apps.

Among the numerous commercially available T2D apps, only a limited number have been formally evaluated in terms of their impact on user engagement outcomes (and published in peer-reviewed journals) or have regulatory clearance [16]. Engagement with digital health interventions, such as T2D apps, has been defined as both an objective measure of use, such as the amount, frequency, duration, and depth of the app accessed, and a subjective experience characterized by attention, interest, and affect [17]. According to Kebede et al [18], in 2018, the most commonly used apps across English- and German-speaking countries included mySugar, MyFitnessPal, OneTouch Reveal, and accu-chek. Most of these apps included self-monitoring and feedback features to track blood glucose levels and keep a diary of dietary intake and physical activity. More recently, however, new apps have been developed that seek to integrate these features; one of them is the Gro Health app, which is the focus of this study. The Gro Health app was selected among other apps because its content considers behavioral change evidence and is endorsed by real-world outcomes reported in 2 studies [4,19], which made it an appropriate app for further research from a behavioral lens.

### The Gro Health App

The Gro Health app is an evidence-based behavior change platform consisting of a dedicated website and an app developed by DDM (previously known as Diabetes Digital Media). The app supports diabetes management by addressing 4 key pillars of health: nutrition, mental well-being, sleep, and physical activity [20]. In terms of nutrition, the app provides nutrition programs, resources, and meal plans personalized to the disease, budget, dietary preferences, and cultural and social norms. In addition, it includes features such as blood glucose tracking, a food diary to track macro- and micronutrients, lifestyle education guides, peer support in a moderated community, and behavior change coaching from health coaches. Within the nutritional programs available, the app offers a Low-Carb program, which is a 12-session, educational behavior change intervention for glycemic control and weight loss for adults with prediabetes and T2D. This program had been evaluated through a real-world 12-month outcomes study that demonstrated improvement in terms of glycemic control and weight loss among participants who completed 9 core lessons of the program [4]. Despite these established benefits, further research is required to better understand the factors that influence the adoption of a healthy diet using the app from a behavioral perspective; a more comprehensive understanding of such factors could be achieved through a systematic theory-based approach using BCW.

### The BCW Approach

**Figure 1:**
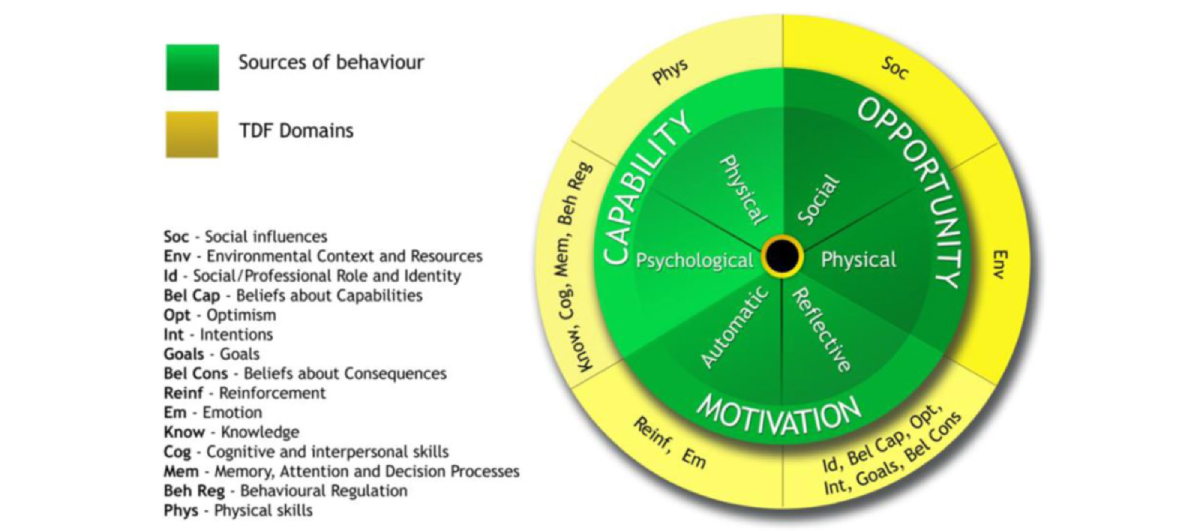
Capability, Opportunity, Motivation, and Behavior model and Theoretical Domains Framework (TDF) domains, from Atkins et al [24] which is published under the Creative Commons Attribution 4.0 International License (CC-BY) [26].

The BCW approach was developed through the synthesis of 19 frameworks of behavior change to aid behavior change intervention design and to improve the process of intervention evaluation and theory development [21]. The BCW approach includes 4 behavioral science tools and demonstrates how they interlink and can be applied to understand behavior and design behavior change interventions. The 4 tools include the Capability, Opportunity, Motivation, and Behavior (COM-B) model, the Theoretical Domains Framework (TDF) [22], the BCW, and the Behavior Change Techniques Taxonomy (BCTTv1). The COM-B model and TDF guide the understanding of behavior, whereas the BCW and BCTTv1 guide the development and content of behavior change interventions [23]. By using COM-B, researchers can better understand behavior in the context where it occurs, determining which aspects in terms of capability (psychological and physical), opportunity (physical and social), and motivation (automatic and reflective) act as enablers or barriers to behavior change. The TDF provides a more granular understanding of the COM-B components (Figure 1 [24]) by further detailing the factors that influence behavior. The COM-B and TDF identify what needs to shift for the desired behavior to be achieved and therefore what to target in an intervention, whereas the BCW identifies intervention functions and supporting policies that are likely to be effective in bringing about change [21].

An expert consensus allows the mapping of the COM-B components and TDF domains with the BCW intervention functions. This leads to the next step of the BCW approach, which consists of identifying intervention content in terms of which BCTs best serve intervention functions and the appropriate mode of delivery to implement the intervention [21]. The BCTTv1 serves as a standardized language for describing distinct BCTs, which serve as the active ingredients in interventions; it lays the foundation for the reliable and systematic specification of behavior change interventions [15]. The appropriateness of an intervention and BCT can be assessed by applying the affordability, practicability, effectiveness/cost-effectiveness, acceptability, side effects/safety, and equity (APEASE) criteria, which acknowledge contextual factors that may influence implementation and have been previously used in the development of health apps to ensure app design simplicity and user-friendliness [25].

### Objectives and Research Questions

The adoption of a healthy diet is essential for ensuring a better prognosis; however, it remains challenging for patients with prediabetes and newly diagnosed T2D as well as for patients with a long-standing diagnosis. The Gro Health app has shown promise as a tool for users to achieve better T2D self-management by incorporating features and BCTs, such as meal plans, information sheets, and a recipe library [4]. However, further research is needed to better understand how users engage with the app’s features and then offer strategies for improvement.

Using the BCW approach, this study aimed to identify the enablers and barriers to adopting a healthier diet before and after the use of the Gro Health app in patients with prediabetes, newly diagnosed T2D, and long-standing T2D. A comparison of the enablers and barriers before and after the use of the Gro Health app can help distinguish the features of the app that contribute to the adoption of a healthy diet. In addition, patients with recently diagnosed T2D and those with long-standing T2D are evaluated in separate studies to identify similarities and differences in terms of patient acceptability of app features and to propose BCTs that are best suited to address the specific engagement barriers of each group.

This study used the BCW approach to answer 3 key research questions (RQs):

- RQ1: per COM-B and TDF, what are the enablers and barriers to adopting a healthier diet in patients with recently diagnosed T2D or those with prediabetes before and after the use of the Gro Health app?
- RQ2: per COM-B and TDF, what are the enablers and barriers to adopting a healthier diet in patients with long-standing T2D, diagnosed for >1.5 years, before and after the use of the Gro Health app?
- RQ3: Do barriers and enablers to adoption differ between patients with recently diagnosed T2D (<12 mo) and those with long-standing (>1.5 y) T2D?
- RQ4: What BCTs could support the Gro Health app, or any other app developed specifically for patients with T2D, in overcoming barriers to promote the adoption of a healthier diet in each patient subgroup?

## Methods

### Study Design

Two studies were conducted. The first was a semistructured qualitative interview study encompassing in-depth interviews with patients with newly diagnosed T2D (<1 y after diagnosis), conducted between June and July 2021. The second study was also a semistructured qualitative interview study focusing on patients with a long-standing diagnosis (>1.5 y after diagnosis), conducted between May and June 2022.

Semistructured qualitative interviews were selected in both studies because little is known about the behavioral enablers and barriers to adopting a healthier diet using an app in patients with newly diagnosed and long-standing T2D. This study design allowed the researchers to probe in greater detail to obtain better insight into the required content of BCTs to support the Gro Health app (in support of RQ4).

### Recruitment

#### Study 1: Patients With Newly Diagnosed T2D

In this study, participants were eligible to participate if they (1) were newly diagnosed (<1 y) with T2D or prediabetes, (2) had received a recommendation from their health care provider to adopt a healthier diet, (3) were aged >18 years, (4) were fluent English speakers, (5) owned a smartphone, and (6) were willing to interact with an app for 2 weeks to adopt a healthier diet.

A total of 16 participants were recruited via a Diabetes UK advertisement to their users (email and communities) and diabetes-related social media groups (eg, via Facebook) in the United Kingdom, with 8 participants completing the interviews. The advertisement was live and reposted several times over 2 months (June to July 2021), offering 1-year access to the Gro Health app and inviting patients with newly diagnosed (<12. mo) T2D or prediabetes who were advised by their health care team to adopt a healthier diet; the patients were asked to use the app for 2 weeks and share their experiences during interviews. A QR code directed potential participants to a screening questionnaire embedded in the University College London (UCL) REDCap (Research Electronic Data Capture; Vanderbilt University) safe haven to guarantee anonymity. For participants meeting the aforementioned eligibility criteria, basic demographic information (eg, age and gender) and patients’ specific diagnosis (T2D or prediabetes; Table S1 in Multimedia Appendix 1) were collected. Participants consented to the study by reading and signing an e-document containing the necessary information, after which an email was automatically sent with instructions for the 2-week app use and interview scheduling.

#### Study 2: Patients With a Long-Standing Diagnosis

In this study, participants were eligible if they had been diagnosed with T2D for >1.5 years, and met screening criteria 2 to 6 given in the *Study 1: Patients With Newly Diagnosed T2D* section. The 1.5 years postdiagnosis criterion was defined as the minimum period between initiating study fielding and the time of initial T2D diagnosis to ensure that patients would have been diagnosed before the COVID-19 pandemic (before March 2020), thus minimizing the impact that the pandemic could have had on patient diagnosis and follow-up.

In this study, a web-based advertisement was posted via the on the Diabetes UK Twitter account to recruit potential participants between May and June 2022, offering 6-month access to the Gro Health app. Five hundred respondents expressed initial interest and completed a web-based REDCap survey. Participants were included or excluded based on the responses gathered. In addition, as part of the survey, participants who met the inclusion criteria were asked to provide demographic information such as age, gender, employment status, and educational background; this information was used to select eligible participants while ensuring that there was a balanced distribution in terms of age and gender. Finally, the recruited and interviewed sample (n=15) included participants who had been diagnosed with T2D, met the inclusion criteria, and equitably represented different age and gender groups (Table S1 in Multimedia Appendix 1). Participants who completed the interview were offered £10 (US $12.60) compensation by UCL, as well as extended free access to the app for a total of 6 months, as a token of appreciation for their time.

### Procedure

In both studies, eligible participants were granted free access to the Gro Health app through a voucher provided by the app developers and engaged in a 2-week app use period. The duration of these studies was aligned with the duration of a prior study evaluating a T2D app, which lasted 4 weeks [27], but further reduced to 2 weeks considering the time limitations for fielding in the case of this research project.

Semistructured interviews were conducted via the web by the researchers, owing to the geographic distance between the researcher and participants (located around the United Kingdom) and, to a lesser extent, the COVID-19 restrictions; interviews were held via Teams (Microsoft Corporation). Interviews lasted between 30 and 45 minutes were audio recorded and transcribed verbatim. The participants provided consent to be interviewed and audio recorded before data collection. Upon completion of the interview, participants could continue using the app for the duration of the voucher, which lasted 1 year in study 1 and 6 months in study 2.

### Measures

The interview schedule in both studies was organized into 3 sections, which comprised COM-B– and TDF-aligned questions along with exploratory questions (Table 1), which are designed to explore participants’ barriers and enablers in adopting a healthier diet before and after app use. The first section explored participants’ lifestyle choices after diagnosis and before using the app, which provided information on dietary habits, as well as prior enablers and barriers to adopting a healthier diet. Patients with long-standing T2D were further prompted to discuss prior dietary experiences because they had been living with T2D for a longer period. The second section focused on the participants’ experiences during the 2-week period using the app, assessing their perception of the features included in the app and how they perceived these had supported them, or not, in adopting a healthier diet. The third, shorter section of the interview probed participants’ willingness to continue using the app after the 2-week period and if participants would recommend the app to other patients with T2D.

Semistructured and open-ended questions allowed the exploration of the main COM-B and TDF components in a structured manner while ensuring that specific topics could be further explored in detail. This is consistent with the approach used in other studies that evaluated apps for diabetes self-management and typically used semistructured interviews. The author created questionnaires to assess criteria such as app usability, acceptability, and behavioral impact [28]. The full interview schedules can be found in Multimedia Appendix 1.

**Table 1:** Example questions from the interview schedules.

Interview section and example questions		COM-B^a^ component		TDF^b^ domain
**Eating habits before engaging with the app**				
	When where you diagnosed and who diagnosed you?	Exploratory	Exploratory	
	What were the main recommendations given to you by your doctor or health care team?	Psychological capability	Knowledge skills	
	Before using the app, what were your main struggles when trying to adopt a healthier diet?	Reflective motivation	Beliefs about capabilities	
	In particular, what changes have you made regarding your nutritional habits?	Psychological capability	Behavioral regulation	
**Experience engaging with the app**				
	Which features of the app did you use more often?	Exploratory	Exploratory	
	What made these features particularly useful to you?	Reflective motivation	Beliefs about consequences	
	Have you changed anything in your eating routine since using the app?	Psychological capability	Behavioral regulation skills	
	Any particular aspect of using the app that preempted you from changing your eating routine? Or external aspects not discussed so far?	Physical opportunity	Environmental context and resources	
**Future outlook**				
	In the mid to long term, are you planning to keep using the app?	Reflective motivation	Intentions	
	Is there anything else that we have not covered so far that you would like to discuss?	Exploratory	Exploratory	

^a^COM-B: Capability, Opportunity, Motivation, and Behavior.

^b^TDF: Theoretical Domains Framework.

### Data Analyses

#### Analyses of Interviews

In both studies, interview transcripts were analyzed in a stepwise approach: a deductive framework analysis was followed by inductive thematic analysis, based on the guidance for data analysis using COM-B and TDF provided by Atkins et al [24].

##### Step 1: Deductive Framework Analysis

To address RQ1 and RQ2, the data were coded against COM-B and TDF to generate the framework for content analysis. In study 1, 2 transcripts were reviewed by a second coder, and an agreement rate of 92% was obtained. In study 2, 2 pilot transcripts were reviewed by a second coder, reliability checks were carried out on the transcripts, and an agreement rate of 82.46% was achieved; these pilot transcripts served to develop a codebook that was used to guide subsequent coding.

##### Step 2: Inductive Thematic Analysis

To further understand the enablers and barriers influencing behavioral change in this context, the data were also assigned an inductive code. Data were analyzed following Braun and Clarke’s [29] thematic analysis process: (1) transcripts were read and reread to allow content familiarization; (2) text relevant to the RQ was highlighted in Word (Microsoft Corporation), coded to COM-B components and TDF domains, and pasted into Excel (Microsoft Corporation); (3) within the identified TDF domains, similar codes were grouped into potential themes and classified as either a barrier or an enabler to the target behavior before or after app use; (4) all themes and domains were reviewed to ensure that all relevant data were analyzed and corresponded to the COM-B components; and (5) main domains or components and themes were tabulated and ranked. To identify which theoretical model domains were the main barriers to and facilitators of healthier diet adoption, both before and after the use of the Gro Health app, they were arranged according to the incidence of mentions and then by the frequency of respondents who stated them.

In study 1, the analysis was validated by a second coder who read 20% of the transcript data, with a high level of code agreement (92%) [30]. In study 2, the codes and grouping in subthemes were checked with a second coder to ensure the reliability and validity of coding; discrepancies were discussed until agreement was reached.

#### Comparison of Findings Between Studies

To address RQ3, the enablers and barriers to adopting a healthy diet before and after using the app were qualitatively compared by the first author to assess how these differed between patients with recently diagnosed T2D and those with long-standing T2D.

#### Identifying Intervention Strategies Using the BCW

To address RQ4, the enablers and barriers (coded to COM-B and TDF) were mapped against BCTs using BCTTv1 according to the approach described by Johnston et al [31] to facilitate linking the TDF domains to the relevant BCTs. This allowed the identification of potential types of BCTs that may optimize Gro Health app content. The APEASE criteria, as described by Michie et al [21], were applied to the identified BCTs to determine their appropriateness for implementation by app users and developers (for BCTs that require app design or content changes per se).

### Data Exclusion

In study 1, 2 patients decided not to use the app after accessing it for the first time. For these participants, the data analysis focused on the first interview section “eating habits before engaging with the app.”

### Ethical Considerations

Ethics approval was granted for both studies by the Departmental Research Ethics Committee of the UCL (20027/001 and 22417.001). Personal identifiers were removed, and the data were stored securely.

## Results

### Enablers and Barriers to Adopting a Healthier Diet

The enablers and barriers influencing the adoption of a healthier diet were identified across the COM-B components and TDF domains before and after the participants engaged with the app.

#### Before Using the Gro Health App

##### Overview

**Figure 2:**
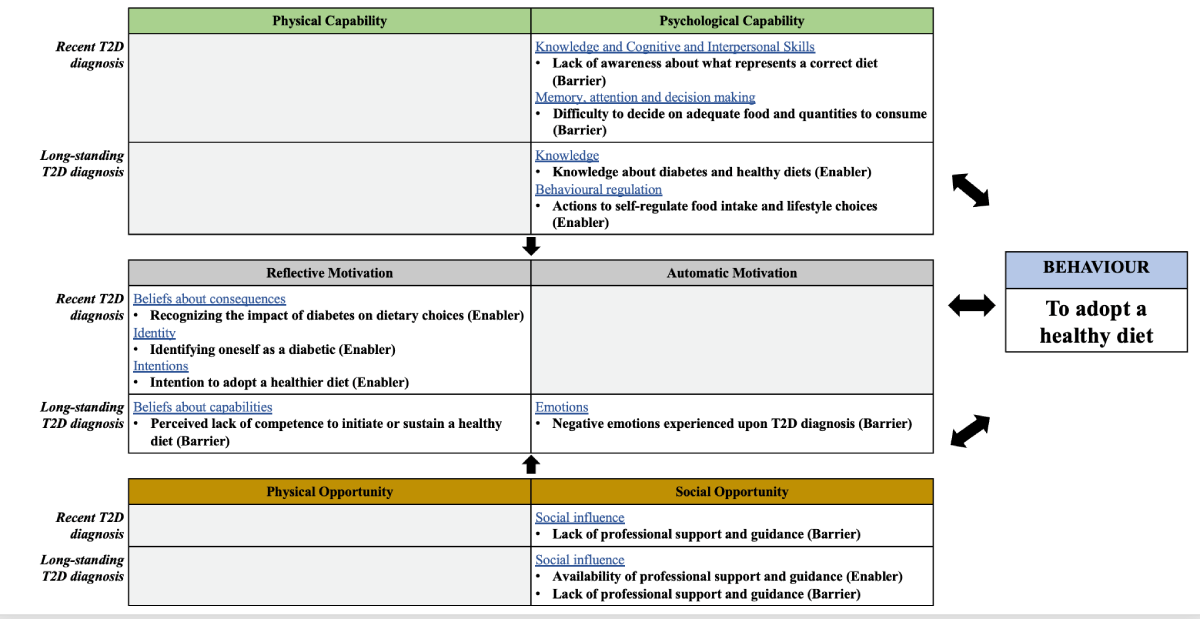
Map of the main themes before using the app in both patient groups, indicating Capability, Opportunity, Motivation, and Behavior (COM-B; overarching) themes; Theoretical Domains Framework (TDF; secondary) themes; and inductive subthemes. T2D: type 2 diabetes. Overarching COM-B themes are indicated by shaded boxes, and TDF secondary themes are indicated by blue underlined text. The inductive themes are indicated in bold. Word in brackets indicate whether each subtheme was identified as an enabler or a barrier. Arrows show the COM-B interactions. The gray boxes correspond to COM-B components for which no main themes were identified.

Six core themes were identified from the interviews in each study, corresponding to the most frequently mentioned statements by participants, identified as either enablers or barriers, and categorized according to the COM-B model and the TDF (Figure 2). Tables S2-S5 of Multimedia Appendix 2 show the rank order and main themes of the enablers and barriers identified for patients with recently diagnosed T2D and those with long-standing T2D before using the Gro Health app, respectively. As indicated by the arrows in Figure 2, the themes within the COM-B components of capability, opportunity, and motivation interact to generate the behavior in scope, which in turn influences these components (ie, enacting the behavior can in turn alter capability, motivation, and opportunity).

##### Enablers to Adopting a Healthy Diet in Patients With Recently Diagnosed T2D: Reflective Motivation

The main enablers identified were coded within this COM-B component, among which, recognizing the impact of diabetes on dietary choices (TDF domain: *beliefs about consequences*) was identified as a key enabler to adopt a healthy diet:

Well, you have to learn how to manage it [diabetes], because I think once you’re diabetic, the doctor says you’re diabetic for life.

P2

In addition, identification as a person with diabetes or prediabetes in need of help (TDF domain: *Identity*) was identified as a catalyst for behavior change among various patients, whereas some of the patients stated that they had taken a conscious decision to adopt a healthy diet (TDF domain: *intention*):

And it’s in my hands now. And so just doing that it’s a case of right, I’m going to educate myself and get a bit healthier.

P4

##### Barriers to Adopting a Healthy Diet in Patients With Recently Diagnosed T2D: Psychological Capability

Barriers coded within this COM-B component comprised three of the 14 TDF domains: (1) *knowledge*; (2) *cognitive and interpersonal skills;* and (3) *memory, attention, and decision-making.*

Within the TDF domain of *knowledge*, patients cited that they had difficulty understanding what constitutes a correct “low-carb” diet:

What is the right low carb diet? What percentage of my diet should be proteins, carbs, and fats? I had no idea.

P4

In terms of *cognitive and interpersonal* *skills*, patients mentioned their struggle to adopt such a diet:

It’s not that I don’t know how to diet, I’ve done it many times before, but not as a for life kind of thing, just for a few weeks or months.

P2

In terms of the TDF domain of *memory, attention, and decision-making*, patients who had lacked the ability to find missing information resorted to web-based search engines and social media and found the amount and diversity of information overwhelming. The lack of understanding on which “low-carb” diet to choose or what to trust was outlined as a barrier to adopting a healthy diet:

So I go off, and I’m reading just about every website and thing I can find that will sort of advise me. Now I find by doing that, you get quite a bit of conflicting information. So that just generates more questions.

P14

###### Barriers to Adopting a Healthy Diet in Patients With Recently Diagnosed T2D: Social Opportunity

Within this COM-B component, the identified barriers correspond to the TDF domain *social influences*. The lack of support from health care professionals was unanimously reported as the main reason for not being able to adopt a healthier diet. Most patients reported only being told the diagnosis, without further assistance:

I was left to my own luck. I received a call from my GP saying I’ve looked into your tests, and you have Diabetes. There was no “you should stop eating this or cut down on that,” no nothing.

P2

##### Enablers to Adopting a Healthy Diet in Patients With Long-Standing T2D: Social Opportunity

Within this COM-B component and the TDF domain *social influences,* the main enabler was the “availability of professional support and guidance,” provided mostly by general practitioners and diabetic nurses, to help patients with T2D better understand the nutritional changes that they needed to make and sustain.

###### Enablers to Adopting a Healthy Diet in Patients With Long-Standing T2D: Psychological Capability

In terms of this COM-B component and the TDF domain *knowledge*, the central theme was the “knowledge about diabetes and healthy diets” that participants had, which enabled them to initiate or maintain a diet; this knowledge was acquired by accessing books, flyers, web-based resources, and courses. In terms of the TDF domain *Behavioral regulation*, the key enabler identified corresponded to the “actions that participants took in terms of self-monitoring their food intake”:

For me it’s about, in my head, always calculating how much carbohydrates I’ve had so far, to determine what I will have.

P6

##### Barriers to Adopting a Healthy Diet in Patients With Long-Standing T2D: Social Opportunity

Within this COM-B component, and in terms of the TDF domain *social influences,* the “lack or limited professional support” was often mentioned as the main barrier to adopt a healthier diet:

I was just told to check labels. The diabetic nurse just said anything under 5g of sugar you can take, anything over 5g don’t eat that. That was it. That was the sum and total of the support.

P9

###### Barriers to Adopting a Healthy Diet in Patients With Long-Standing T2D: Automatic Motivation

A key barrier that was coded within the TDF domain *emotions* comprised the “negative emotions experienced upon diagnosis,” such as anger, shock, or surprise, which may have temporarily hindered patient motivation to change nutritional habits.

###### Barriers to Adopting a Healthy Diet in Patients With Long-Standing T2D: Reflective Motivation

Within the TDF domain *beliefs about consequences*, the “perceived lack of competence to initiate or maintain a healthy diet” was often mentioned as a barrier for behavior change:

I also personally don’t have the capability for eating meat. That really seems to be required on things like Atkins and keto diets. I eat meat, but I don’t eat it in the quantities that they recommend.

P14

#### After Using the Gro Health App

**Figure 3:**
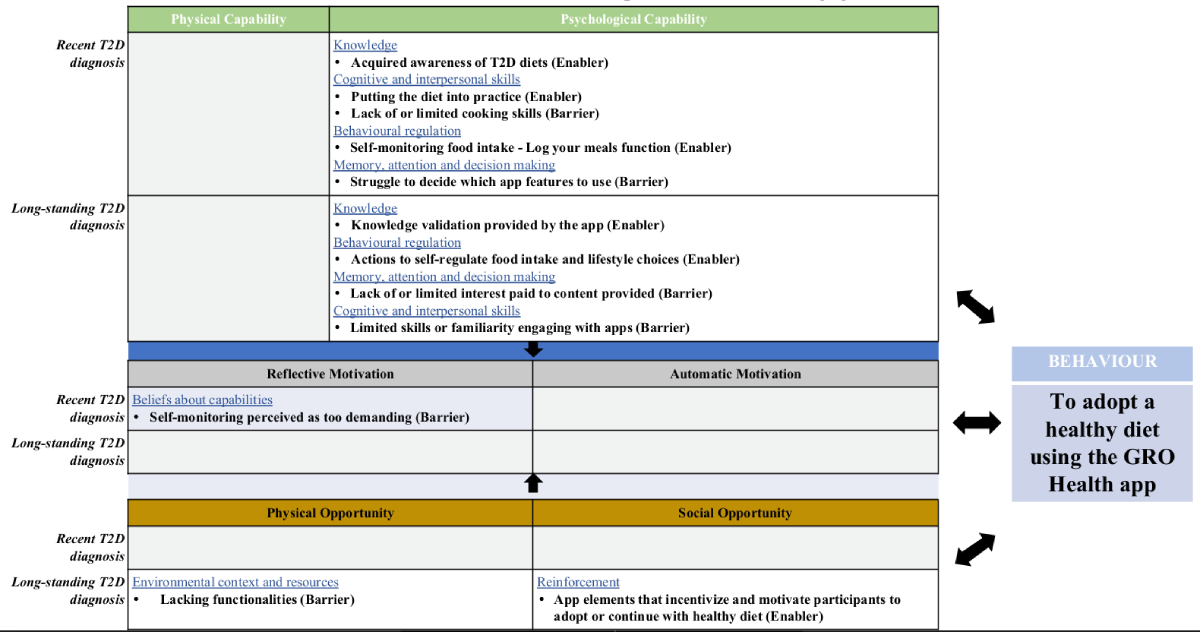
Map of the main themes after using the app in both patient groups, indicating Capability, Opportunity, Motivation, and Behavior (overarching) themes, Theoretical Domains Framework (secondary) themes; and inductive subthemes. T2D: type 2 diabetes.

After engaging with the app, 6 core themes were identified in each study as the most common enablers or barriers (Figure 3). Tables S6-S9 in Multimedia Appendix 2 show the rank order and main themes of the enablers and barriers identified for patients with recently diagnosed T2D and those with long-standing T2D after using the Gro Health app, respectively.

##### Enablers to Adopting a Healthy Diet in Patients With Recently Diagnosed T2D: Psychological Capability

The main enablers were categorized within this COM-B component; among these, patients reported that using the app increased their knowledge of an ideal T2D diet and clarified prior dietary misconceptions (TDF domain: *knowledge*):

I always thought I would have to stop eating a bunch of things I can’t live without, but then I’ve learned is more about portion control and compensations, and there is no such a thing as prohibited foods.

P17

In addition, all patients reported translating their acquired knowledge into new skills (TDF domain: *cognitive and interpersonal skills*), actively lowering their carbohydrate consumption, managing portion sizes, including more fruits and vegetables in their meals, and making new recipes.

Patients unanimously mentioned using at least 1 self-monitoring tool for tracking progress with the app, with food logging being the most frequently used**,** demonstrating the TDF domain *behavioral regulation*. Increased food intake self-monitoring was cited as an important driver for adopting a healthier diet:

Yes, logging my meals is very helpful. With that I can plan myself, because it shows me how much of each nutrient I have eaten and what is the ideal amount for the day.

P11

##### Barriers to Adopting a Healthy Diet in Patients With Recently Diagnosed T2D: Psychological Capability

Most of the identified barriers to the adoption of a healthier diet using the app were within this COM-B component, particularly within the TDF domains of (1) *memory, attention, and decision-making* and (2) *cognitive and interpersonal skills*.

Although all participants reported that their dietary capabilities improved using the app, many experienced, at some point in time, a cognitive overload (TDF domain: *memory, attention*, *and decision-making)* because of the considerable amount of information available simultaneously, which made navigation of the app cumbersome and counterintuitive:

It can get quite busy with all the articles and videos...there is no order for you to follow and sometimes I would feel like there is so much going on at the same time here.

P9

In addition, some participants cited having difficulty understanding how to measure food, highlighting a lack of cognitive skills, which limited the use of the food logging tool and reduced motivation. Similarly, patients also complained about not finding suitable replacements and stated that they did not have the skills needed to cook some of the proposed recipes, presenting a barrier to diet change.

###### Barriers to Adopting a Healthy Diet in Patients With Recently Diagnosed T2D: Reflective Motivation

Some patients found the app’s self-monitoring tool *log your meals* demanding or difficult to use, stating that it made them less willing to log meals after a few days:

When I eat something I can’t find on the app’s list to log, I just don’t do it. It’s too much work to try to guess what is on the food you ordered. But I do admit that not putting on the app makes me lose track of things and probably eat more carbs than I should.

P14

This highlighted a barrier within the TDF domain *beliefs about capabilities*, with some participants feeling less confident about dieting, whereas others believed that they had slipped further out of a healthier diet.

##### Enablers to Adopting a Healthy Diet in Patients With Long-Standing T2D: Psychological Capability

In terms of the enablers within this COM-B component, the main TDF domains identified include *knowledge* and *behavioral regulation* (Figure 3), consistent with the findings observed before using the app. In terms of *knowledge*, the app mainly validated preexisting information that participants had previously encountered to further their understanding of T2D and its dietary requirements. With regard to *behavioral regulation,* the app allowed users to more broadly undertake various actions to self-regulate their food intake, using elements such as the *meal plans* and *log your meals* functionalities, and this helped participants to better plan their meals and self-monitor their food intake:

I went back to monitoring what I cooked for a while, but altered the proportions and the amounts. And from that I started to realize that carbs were too high and needed to come down.

P7

###### Enablers to Adopting a Healthy Diet in Patients With Long-Standing T2D: Automatic Motivation

An additional enabler often identified corresponds to the TDF domain *Reinforcement*, which encompassed the combination of “app elements that incentivized and reinforced patients in their conviction to adopt or continue with a healthy diet”:

The range of the planning of the weekly plans plus the variety of recipes make it more fun, it’s more motivating.

P3

##### Barriers to Adopting a Healthy Diet in Patients With Long-Standing T2D: Physical Opportunity

Most of the barriers identified fell within this COM-B component, particularly within the TDF domain *environmental context and resources*. The “lacking functionalities” was the most recurring theme mentioned by patients, in particular the lack of meal options in the weekly planners and ingredients in the *log your meal* function and the inability to synchronize data exchange with other apps or blood glucose meters.

#### Comparison of Enablers and Barriers Between Patients With Recently Diagnosed T2D and Those With Long-Standing T2D

The comparison between the 2 patient groups (Table 2) showed that in proportion to the total number of statements coded, the patients with recently diagnosed T2D reported more barriers to adopting a healthy diet than patients with a long-standing diagnosis before using the app (69% vs 47%, respectively). Conversely, after using the app, patients with recently diagnosed T2D reported more enablers than barriers compared with patients with a long-standing diagnosis (81% vs 63% respectively).

In terms of TDF domains, there were differences in the enablers and barriers before using the app between the 2 patient groups (Figure 2), with no overlapping domain between groups, except for *knowledge*. The *knowledge* domain was noted as a barrier for recently diagnosed patients, in contrast to long-standing diagnosis patients who referred to it as an enabler. In contrast, when using the Gro Health app, both patient groups coincided in terms of most of the TDF domain enablers (*knowledge* and *behavioral regulation*) and barriers (*memory, attention*, *and decision processes* and *cognitive and interpersonal skills*), although the underlying themes differed. For instance, referring to the barriers within the TDF domain *memory, attention and decision processes* patients with a recent diagnosis referred to their “struggle to decide which app features to use,” whereas patients with a long-standing diagnosis expressed a “lack of/limited interest paid to the content provided.”

**Table 2:** Relative frequency of enabler and barrier statements before and after using the app between patients with recently diagnosed type 2 diabetes (T2D) versus those with long-standing T2D.

Patient group	Before using app (%)				After using Gro Health app (%)		
	Enablers	Barriers	Total	Enablers		Barriers	Total
Recently diagnosed with T2D	31	69	100	81		19	100
Long-standing T2D	53	47	100	63		37	100

#### Recommended BCTs

##### BCTs to Support the Gro Health App in Overcoming Barriers

A mixture of enablers and barriers to adopting a healthier diet using the Gro Health app were identified across various TDF domains and represented targets for behavioral change interventions. Working through the BCW intervention development process, including the APEASE criteria, the most appropriate BCTs were identified to address the main barriers identified among patients with recently diagnosed T2D (Table S10 in Multimedia Appendix 2) and those with long-standing T2D (Table S11 in Multimedia Appendix 2). The APEASE criteria, as described by Michie et al [21], were applied to the identified BCTs to determine their appropriateness for implementation by app users and developers (in the case of BCTs requiring app design or content changes per se).

Regarding the recommended BCTs, both studies suggested the use of the following: “restructuring the physical environment,” “instruction on how to perform a behavior,” and “conserving mental resources”; however, the studies differed in terms of other BCTs to consider. Patients with a recent diagnosis were suggested BCTs that were predominantly aimed toward addressing *beliefs about capabilities* barriers (eg, “demonstration of the behavior”), whereas those with a long-standing diagnosis were suggested BCTs to enhance their skills and prompt them to engage with the app.

##### Comparison of the Recommended BCTs Versus Existing BCTs in the Gro Health App

The proposed BCTs from both studies were further compared with the BCTs already included in the Gro Health app. The individual BCTs in the Gro Health app were coded against the BCTTv1, resulting in 33 BCTs being identified out of the 93 in the BCTTv1 (refer to Table S12 in Multimedia Appendix 3); comparing the outcomes of this coding assessment with the proposed BCTs (Table S13 in Multimedia Appendix 3) suggested that most of the latter (10/11, 91%) were not entirely new BCTs and likely served to supplement the existing BCTs within the app, whereas a minority of the BCTs (1/11, 9%) were not currently leveraged by the app and represented a new approach toward the target behavior change.

## Discussion

### Principal Findings

#### Overview

This qualitative study identified key enablers and barriers to adopting a healthier diet using an app (Gro Health) among patients with recently diagnosed T2D and those with long-standing T2D. We outlined the similarities and differences between patient groups and proposed BCTs to address these barriers. The main enablers for patients with recently diagnosed T2D in terms of TDF domains were *knowledge, cognitive and interpersonal skills*, and *behavioral regulation*, whereas the main barriers were *memory, attention, and decision processes*; *cognitive and interpersonal skills*; and *beliefs about capabilities*. For patients with long-standing T2D, the main enablers were similar and included TDF domains *knowledge* and *behavioral regulation.* However, in contrast to patients with a recent diagnosis, these patients identified *reinforcement* as a key enabler. The main barriers were also similar to those of recently diagnosed patients and included *memory, attention*, *and decision processes* and *cognitive and interpersonal skills*. However, for this patient group, *environmental context and resources* were identified as key barriers. In further comparing findings between patient groups, both groups reported more enablers than barriers to performing the target behavior when using the app, with overlap in most of the enablers and barriers encountered. Consequently, BCTs such as *restructuring the physical environment*, *instruction on how to perform a behavior* and *conserving mental resources* were recommended as relevant BCTs to address key barriers in both groups.

#### Enablers and Barriers to Adopt a Healthier Diet Before Using the Gro Health App

This study’s results are consistent with the findings of other studies that reported enablers and barriers to adopting healthier diets among patients with T2D, with or without the support of apps. Some of the main enablers identified before using the app, namely, *behavioral regulation*, *knowledge*, and availability of low-carbohydrate food options, are consistent with the findings by Cradock et al [32], who identified various facilitators of healthy diet behaviors among patients with T2D in Ireland (with similar demographics to this study), such as home and work food planning, education to assist in adopting and maintaining a healthy diet, and availability of healthy food choices when shopping.

In terms of barriers, this study identified the lack of professional guidance and support in providing the information patients needed as a prominent barrier to behavior change. This is consistent with the findings by Hynes et al [33], whereby the absence of trusted guidance from health care professionals impaired the patients’ self-management skills and motivation to implement lifestyle modifications. Pikkemaat et al [7] reached the same conclusion but also connected the resultant lack of structured primary care education to low self-efficacy, low self-confidence, increased stress, and feelings of loneliness among patients with a new diagnosis. This also aligns with the negative emotions reported by the study participants, particularly in patients with long-standing T2D.

#### Enablers and Barriers to Adopting a Healthier Diet Using the Gro Health App

In terms of the main enablers of adopting a healthier diet using the Gro Health app, patients in both studies unanimously considered the information on the Gro Health app to be both trustworthy and credible, which improved their knowledge and self-management skills. This finding concurs with the results of a systematic review of 28 studies evaluating the adoption of T2D apps by patients [34]. *Knowledge* is consistently reported as an enabler, particularly information about T2D, new insights into self-management, and the latest research findings [34,35]. In addition, elements considered within *behavioral regulation*, such as the log your meals and weekly meal planning functions, were praised by both patient groups, consistent with the findings by Trawley et al [36], who also identified these features among the preferred and most useful ones within T2D apps. Finally, with regard to *reinforcement* among patients with a long-standing diagnosis, the benefits of prompts or reminders are documented to a lesser extent than those of the aforementioned enablers, but Jeffrey et al [37] also identified weekly reminders supporting patient self-management as useful features for app users.

By contrast, with regard to the barriers to performing the target behavior, this study reports barriers similar to those identified in the literature, while also identifying some less frequently encountered ones. Common barriers in terms of *environmental context and resources* include “lacking functionalities” and the “perceived complexity of the app design,” which resonate with other studies that identify these app-specific elements as common barriers to engaging with a T2D app [34,37].

Within the domains of *memory, attention*, *and decision processes,* the cognitive overload reported by patients with a recent diagnosis due to the app providing too much simultaneous information. This has also been reported by Katz et al [38] in their evaluation of type 1 diabetes apps, highlighting the importance of reducing cognitive demands in terms of use requirements. In addition, the “lack of/limited interest paid to the content provided” among patients with long-standing T2D is consistent with previous findings, suggesting that if patients are confident of their lifestyle management decisions without using apps and do not perceive a benefit from T2D apps, they are unlikely to use them for T2D self-management [34,36]. Highlighting the added benefits that the Gro Health app may offer, in addition to other self-management initiatives, may be an approach to better engage patients with long-standing diabetes, as these individuals have usually tried various self-management approaches before trying an app. Further research on this topic is recommended.

Finally, in terms of *beliefs about capabilities*, patients with a recent diagnosis considered self-monitoring using the app to be too demanding, whereas in terms of *cognitive and interpersonal skills*, they highlighted the “lack of/limited cooking skills” as a main barrier to adopting a healthier diet. Within the same TDF domain, patients with a long-standing diagnosis mentioned the “limited skills or familiarity engaging with apps” as a key barrier. Altogether, the barriers identified in these domains are consistent with the findings from previous studies that highlight patient self-perception of technological literacy as a key barrier to engaging with T2D apps [37], which can be addressed by providing training on how to use an app [34]. To address these barriers, further understanding of the similarities and differences between patients with recently diagnosed T2D and those with long-standing T2D could be beneficial to inform the customization of the app for specific user requirements.

#### Comparison of Enablers and Barriers Between Patients With Recent and Those With Long-Standing Diagnoses

Differences in terms of the types of enablers and barriers and their respective frequency of mentions were identified between the 2 patient groups before using the app; however, after engaging with it, both patient groups reported more enablers and similar types of enablers and barriers, contrary to the expectations of the researchers. These findings may be explained by understanding the motivational predictors to initiate or maintain T2D dietary changes in each patient group. The 2 studies assessed the predictors of dietary self-care in these populations; among the newly diagnosed group, changes in dietary self-care are associated with perceived self-efficacy, self-evaluation (ie, self-monitoring), and controlled motivational behaviors, which occur when patients are pressured either by their interpersonal environment or by guilt or fear [39] (refer to Multimedia Appendix 4 [38] for a description of the constructs used). In contrast, in patients with long-standing T2D, changes in dietary self-care are mostly associated with self-efficacy and autonomous motivation, that is, behaviors that are self-initiated because they are important to the individual and tie into their values and goal system [40]. The association with self-efficacy in both groups may explain why they concur on enablers such as *knowledge* and *behavioral regulation*, whereas the difference in terms of motivation may partly explain the differences in barriers: among patients with a recent diagnosis, factors related to the TDF domains of *social influence* and *beliefs about capabilities* were identified as key barriers in study 1, whereas among patients with a long-standing diagnosis, factors related to *environmental context and resources* were identified as main barriers for the target behavior.

The similarities and differences identified in terms of enablers and barriers between patient populations lead to the identification of BCTs that may benefit both populations or only 1.

#### BCTs to Support the Gro Health App in Enhancing Enablers and Overcoming Barriers

The proposed BCTs further build on the existing behavioral components included within the Gro Health app. This app includes a relatively high number of BCTs (32 out of the 93 BCTs in the BCTTv1), whereas the average number reported by Priesterroth et al [14] was 7.4 BCTs in other diabetes apps. This implies that the Gro Health app already includes a considerable number of BCTs, although these were not exclusively included to support the adoption of a healthier diet but were considered in a more holistic manner to support various self-management behaviors (eg, physical activity, sleep, and mental well-being). In this context, the BCTs suggested in this study may enhance the app by further tailoring its nutritional self-management content to achieve the target behavior.

The suggested BCTs not only are consistent with those that have been previously identified in studies evaluating T2D apps but also include novel BCTs that have not been often reported in these apps. As reported in other studies [12,14], “self-monitoring of behavior,” “prompts/cues,” and “conserving mental resources” are consistently identified BCTs in T2D apps, which have already been included, to some extent, in the Gro Health app design, likely due to these BCTs having a clear link with diabetes self-management tasks. In contrast, other BCTs suggested in this study, such as antecedents (eg, “restructuring the physical environment” and “avoidance/reducing exposure to cues for the behavior”), shaping knowledge (“instruction on how to perform the behavior”), and repetition and substitution BCTs (“graded tasks”), have either been reported in <10% of T2D apps [14] or not reported at all in previous studies, despite supporting rationale for their inclusion.

The following have been used as constituent BCTs in randomized controlled trials that evaluate interventions to change dietary activity in patients with T2D: “instruction on how to perform the behavior,” “avoidance/reducing exposure to cues for the behavior,” and “graded tasks.” The results of those trials suggest that the presence of these BCTs is associated with reductions in hemoglobin A_1c_, although these results were not statistically significant [32]. Although the study by Cradock et al [32] did not focus on the use of T2D apps, it is indicative of the potential of the aforementioned BCTs to positively impact the adoption of a healthier diet, either by directly influencing dietary activity or by further engaging users with the app. Further studies, ideally using experimental designs, are required to evaluate how these BCTs impact the adoption of a healthier diet using T2D apps to validate their potential.

Although the BCTs described so far are applicable to both groups of patients, certain BCTs may be best suited for one population or the other. For instance, patients with a long-standing diagnosis may benefit more from “prompts/cues” that serve as reminders to adopt a healthier diet, helping them better manage environmental factors such as time restrictions, which in turn may lead them to forget checking the app; in contrast, patients with a recent diagnosis further benefit from BCTs that enhance their *beliefs about capabilities*, such as “feedback on behavior.” This finding has further implications for app developers in terms of the customization and support provided by the apps.

### Implications for Practice

This study identified enablers and barriers, as well as BCTs, to ultimately support patients with T2D in adopting a healthier diet with an app. The enablers identified reemphasize the need for T2D app developers to include app features that provide further knowledge to users regarding T2D and nutrition, allow for self-monitoring and action planning *(TDF domain: behavioral regulation)*, and reinforce prior actions taken by users to adopt a healthier diet. In addition, app developers should be cognizant of particular barriers that patients with recently diagnosed and long-standing diabetes encounter in terms of the app per se (eg, cognitive overload and perceived complexity of app functionalities), the external environment (eg, lacking functionalities), and the skills that users need to have to either use the app or implement its suggestions. The BCTs suggested in this study not only build on those already included in the Gro Health app but also represent new options to consider in terms of app design (eg, including “graded tasks” and “feedback on the outcomes of behaviors” BCTs to encourage engagement with the app). Although the APEASE criteria inform the appropriateness of these BCTs, further validation through additional studies is required to corroborate BCT appropriateness and its impact on the target behavior.

This study also identified the commonalities and differences faced by patients with recently diagnosed T2D versus those with long-standing T2D when it comes to performing the target behavior. The Gro Health app does not currently customize its content according to the duration of diabetes. This implies the need to further customize certain app features to better respond to the differing needs of these patient populations and to address the different barriers they may encounter. The extent to which such customization should be implemented requires further research, given that this study has limitations in terms of the generalizability of its results across populations.

### Limitations

This study has several limitations. The results correspond to a particular app (Gro Health) that was evaluated in a small sample size, potentially limiting the generalizability and applicability of this study’s findings to other apps and the broader population; however, it is worth noting that the overall supply of T2D apps changes continually, which hinders potential comparison to other apps. In addition, it was not possible to monitor app use patterns during the 2-week period; this might have led to social desirability bias among participants during the in-depth interviews when asked about the frequency with which they used the app.

In terms of the BCW approach, the researchers and second coders are trained in the approach and confident in the interpretation of results; nonetheless, there is scope for potential bias during the analysis process, particularly when coding deductively and inductively, and other researchers may have categorized data in a different manner. In addition, the TDF approach also has some inherent limitations. McGowan et al [41] highlighted that the TDF offers a structured approach that may result in findings becoming self-contained within the relevant domains identified, leading to important factors being overlooked. However, this risk has been mitigated in this study by following the guidance of Atkins et al [24] and by conducting an inductive analysis to generate themes considered in relation to the TDF domains. This allowed for the lack of specificity of some TDF domains, such as *cognitive and interpersonal skills*, to be addressed using descriptive inductive coding (eg, “lack of/limited cooking skills” and “limited skills or familiarity engaging with apps”), which ultimately allowed to identify more adequate BCTs.

A final limitation is that this study did not measure actual behavioral change over time as an outcome (ie, the percentage of patients with T2D who adopted or maintained a healthy diet using an app for 6 mo or 1 y). Instead, the enablers identified when using the app and the BCTs already incorporated into the app served as proxies for effectiveness. Despite this limitation, the results from this study provide a foundation for further evaluation of the effect of the suggested BCTs on the behavior in scope.

### Conclusions

Adopting and maintaining a healthy diet is a challenge for patients with T2D, which can partially be addressed by the use of digital apps. This study used the BCW approach to assess the enablers and barriers to adopting a healthier diet using the Gro Health app for patients with recently diagnosed T2D and those with long-standing T2D. The main enablers identified among both populations in terms of TDF domains included *knowledge* and *behavioral regulation*, whereas the main barriers included *memory, attention*, *and decision processes* and *cognitive and interpersonal skills*. Thematic analysis identified key themes that provided additional insights into the specific enablers and barriers (notably, enablers such as “knowledge validation” and self-regulating and self-monitoring actions and barriers including “lacking functionalities” and “struggle to decide which app features to use”) that could be addressed using BCTs. Consequently, BCTs were identified (per the BCTTv1) with the potential to address the key barriers (eg, “restructuring the environment,” “instruction on how to perform a behavior,” and “conversing mental resources”). Findings from this study revealed similar enablers between patients with recently diagnosed T2D and those with long-standing T2D, with slight differences in terms of barriers to performing the target behavior. These results highlight the importance of understanding enablers and barriers in patients with T2D and suggest that future research is needed to further understand enablers and barriers within patient groups, as well as to implement and validate the effectiveness of the proposed BCTs.

## Appendix

Multimedia Appendix 1 Demographics of study participants.

Multimedia Appendix 2 Rank order of enablers and barriers to adopt a healthier diet for recently diagnosed patients with type 2 diabetes.

Multimedia Appendix 3 Behavior change techniques included in the GRO Health App, based on the Behavior Change Techniques Taxonomy.

Multimedia Appendix 4 Constructs used by Nouwen et al [39].

## Abbreviations

APEASE: affordability, practicability, effectiveness/cost-effectiveness, acceptability, side effects/safety, and equity
BCT: behavior change technique
BCTTv1: Behavior Change Techniques Taxonomy
BCW: Behavior Change Wheel
COM-B: Capability, Opportunity, Motivation, and Behavior
REDCap: Research Electronic Data Capture
RQ: research question
T2D: type 2 diabetes
TDF: Theoretical Domains Framework
UCL: University College London
