# Supplementary material for: Barriers and Enablers to the Adoption of a Healthier Diet Using an App: Qualitative Interview Study With Patients With Type 2 Diabetes Mellitus
Source: JMIR Diabetes. 2023 Dec 19;8:e49097. doi: 10.2196/49097 (PMC10762608; doi:10.2196/49097)
Supplement: Multimedia Appendix 1 [file diabetes_v8i1e49097_app1.docx]

**Table S1.** Demographics of study participants.

| Demographic characteristics | Study 1: Newly diagnosed,  n (%) | Study 2: Longstanding diagnosis, n (%) |
| --- | --- | --- |
| Age (years) |  |  |
| 18-25 | 0 (0) | 0 (0) |
| 26 – 50 | 2 (25.0%) | 5 (33.3%) |
| 51 – 65 | 6 (75.0%) | 5 (33.3%) |
| 65+ | 0 (0) | 5 (33.3%) |
| Gender |  |  |
| Male | 2 (25.0%) | 7 (46.7%) |
| Female | 6 (75.0%) | 8 (53.3%) |
| Trans-gender | 0 (0) | 0 (0) |
| Non-binary | 0 (0) | 0 (0) |
| Other | 0 (0) | 0 (0) |
| Diagnosed condition |  |  |
| Pre-diabetes | 2 (25.0%) | 0 (0) |
| Type 2 diabetes | 6 (75.0%) | 15 (100%) |
| Time post-diagnosis (years) |  |  |
| 0 to 0.5 | 6 (75.0%) | 0 (0) |
| 0.5 to 1.0 | 2 (25.0%) | 0 (0) |
| 1.0 to 1.5 | 0 (0) | 0 (0) |
| 1.5 to 5 | 0 (0) | 5 (33.3%) |
| 6 to 10 | 0 (0) | 4 (26.7%) |
| 11 to 15 | 0 (0) | 2 (13.3%) |
| 15+ | 0 (0) | 4 (26.7%) |
| Employment status | Not measured in this study |  |
| Employed Part time | - | 3 (20.0%) |
| Employed Full time | - | 6 (40.0%) |
| Self-employed | - | 2 (13.3%) |
| Unemployed | - | 1 (6.7%) |
| Retired | - | 3 (20.0%) |
| Student | - | 0 (0) |
| Other | - | 0 (0) |
| Educational background | Not measured in this study |  |
| Elementary | - | 0 (0.0%) |
| High school | - | 2 (13.3%) |
| Bachelors or equivalent | - | 8 (53.3%) |
| Masters or equivalent | - | 1 (6.7%) |
| Doctoral or equivalent | - | 3 (20.0%) |
| Other | - | 1 (6.7%) |

NOTE: Study 2 collected employment status and educational background to ensure a broad patient sample given the larger number of interested participants compared to Study 1.

**Methodology.** Interview schedule – study 1 (newly diagnosed patients).

**INTRODUCTION**

Hello, welcome to this session.

Before we begin, I would like to remind you that all information shared today will be kept anonymous and confidential. Please confirm to me that you all have signed the consent form and are aware of the intent of this research.

**SECTION 1: EATING HABITS BEFORE ENGAGING WITH THE APP**

1. I would like to know a little bit about your eating habits before the use of the app, could you tell me:
   1. What a typical lunch or dinner would look like? And a snack?
   2. How did you feel when you first learnt about your condition?
   3. What were the main recommendations given to you by your doctor/healthcare team? Were you able to act on them? What aspects did you already change before using the app?
2. Before using the app, what were your main struggles when trying to adopt a healthier diet?
3. Were you looking for any tools to support you to adopt a healthier diet? What were those? Did they work? If yes, why / if no why?

**SECTION 2: EXPERIENCE ENGAGING WITH THE APP**

1. Now tell me about your experiences of using the app, what it easy to initiate?
   1. What have you found to be initially useful? Why?
   2. Was it easy to integrate the time demand from the app within your daily routine? Why/why not?
   3. Can you describe your daily/weekly interaction with the app (e.g. at what times, how often)?
   4. Were you able to start implementing anything you learnt from the app? What were the easiest and the most difficult changes or adaptations to your diet?
   5. Which features of the app have you used? Which were the most helpful and why?
   6. Were there things you found less useful, or hard to interact with or not interesting in the app? Why was that?
   7. Have you changed anything in your eating routine since using the app?
   8. Is there any of the lessons that you had that you remember in particular? Why was this lesson important to you? (use articles for reference)

**SECTION 3: FUTURE OUTLOOK**

1. In general, has the app supported you to adopt a healthier diet, how and why?
   1. How does the medium/long term look like for you? Are you planning to keep using the app? If yes, why / if no why?
   2. Add: can you compare how you were feeling about your beliefs about adopting a healthier diet and how are they now?
   3. Separate double questions – first the question and then why is that?
2. And lastly, I would just like to ask you: Is there anything else that we haven’t covered that you would like to talk about?

Thank you so much for sharing all you shared. Your contribution was very important.

**END**

**Methodology.** Interview schedule - study 2 (patients with a longstanding diagnosis).

**INTRODUCTION**

Hello, welcome to this session during which we will discuss your experience testing the app that was provided.

I’m a university researcher and would like to hear about patients the experience of type 2 diabetes participants using the “GRO app” in supporting their adoption of a healthier diet. Just to clarify, I’m not employed by “GRO app”.

There are no right or wrong answers, but I would just ask that you answer the questions as honestly as possible.

Before we begin, I would like to remind you that all information shared today will be kept anonymous and confidential.

Please confirm to me that you all have signed the consent form and are aware of the intent of this research.

The interview will be divided into three parts. The first, talking about your experiences prior to using the app, the second focusing on your experience during the trial period using the app, and the third focusing on your impressions about using the app in the longer term.

Shall we start?

**SECTION 1: EATING HABITS BEFORE ENGAGING WITH THE APP**

To start, we would like to know a little bit about your eating habits before the use of the app.

1. Can you please tell me about your diagnosis:
   1. When were you diagnosed? Who diagnosed you?
   2. How did you feel when you first learnt about your condition?
2. What were the main recommendations given to you by your doctor/healthcare team? Were you able to act on them?
3. What lifestyle changes have you made after your diagnosis of type 2 diabetes?
4. In particular, what changes have you made regarding your nutritional habits?
   1. Has your doctor provided you with information to support you in adopting a healthier diet, such as pamphlet or information during consultation?
      1. If no to the previous question, where did you search for information?
   2. Were you confident with the information you found on adopting a low carb diet?
   3. Could you please tell me what constitutes a typical meal (lunch, snack, and dinner)?
      1. Do you have a partner that helps you or family that supports you to prepare meals?
   4. What aspects of your diet had you already changed before using the app?
5. What have been your main struggles when trying to adopt a healthier diet?
6. What other alternative approaches, aside from using a digital app, had you considered to help you adopt a healthier diet?
   1. What were these approaches?
   2. Did they work? If yes or no, why?
7. Have you thought about using an app before? Why/why not?
   1. What were you thinking about when you decided to use/engage with the app?
   2. Did you expect the app to be able to support you on adopting a healthier diet?

**SECTION 2: EXPERIENCE ENGAGING WITH THE APP**

Now we would like to discuss your experience using the app.

1. Was it easy to initiate using the app?
   1. What features did you find to be initially useful? Why?
   2. What features did you find less useful? Why?
2. Was using the app and its associated time demand easy to integrate within your daily routine? Why/why not?
   1. Can you please describe your daily/weekly interaction with the app (e.g., at what times did you generally use it, how often)?
3. In terms of the nutritional section of the app, were you familiar with the concept of a low carb diet? Was there anything new for you in that sense?
4. Which features of the app did you find more useful to adopt a healthier diet?
   1. What did these features enable you to do?
   2. What made these features particularly useful to you?
   3. Probe: Have you used the recipes/meal plans and cook-along-videos to support you in the adoption of a healthier diet? Were they helpful? In what way?
   4. Probe: have you tried the coach or the communities?
   5. Probe: how did you find the notifications and prompts from the app to support you in the adoption of a healthier diet? Were they helpful? In what way?
   6. Probe: Can you tell me a bit more about the logging your meals and how this is helping you in adopting a healthier diet?
5. Were there features that you found less useful to adopt a healthier diet?
   1. What made these features less useful or difficult to use?
6. Have you changed anything in your eating routine since using the app?
   1. What were the easiest and the most difficult changes or adaptations to your diet? Probe: Why?
   2. If yes, what app feature did you find most useful in terms of changing your eating routine?
   3. If not, please explain why. **Any particular aspect of using the app that pre-empted you from changing your eating routine? Or external aspects not discussed so far?**
   4. What do you think is the most challenging for you to integrate into your diet?
7. Do you remember any particular lessons/learnings?
   1. Why was this lesson important to you?
8. In general, has the app supported you in adopting a healthier diet? How and why?
   1. How does using the app to adopt a healthier diet differ from other things you have tried?
   2. Do you feel the app was able to help you to commit to your goals/motivations for wanting to change your diet? Why / why not?
9. Are there any features that the app didn’t have that you think would be helpful to support you in adopting a healthier diet?

**SECTION 3: FUTURE OUTLOOK**

1. In the mid to long term, are you planning to keep using the app? If yes or no, please explain why.
   1. Outside of this trial period, thinking in the midterm, do you think that there could be anything that would pre-empt you from using the app?
2. Can you compare how you were feeling about adopting a healthier diet before using the app and after?
   1. Has the experience of using the app changed your beliefs about adopting a healthier diet?
3. Do you think that from all your experience you have been having with the app, is this a good tool for people that have been diagnosed diabetes for more than 1.5 years? Why?
4. Are you willing to follow this diet moving forward? How do you think the app will support you on that?
5. Is there anything else that we haven’t covered so far that you would like to discuss?

Thank you very much for sharing your experience with us. Your contribution has been very important.
