# Supplementary material for: Barriers and Enablers to the Adoption of a Healthier Diet Using an App: Qualitative Interview Study With Patients With Type 2 Diabetes Mellitus
Source: JMIR Diabetes. 2023 Dec 19;8:e49097. doi: 10.2196/49097 (PMC10762608; doi:10.2196/49097)
Supplement: Multimedia Appendix 2 [file diabetes_v8i1e49097_app2.docx]

### Multimedia Appendix 2.

**Table S2.** Rank order of enablers and barriers to adopt a heathier diet for recently diagnosed T2D patients – before using the GRO Health app.

| Enablers or Barriers | COM-B | TDF Domain | Rank order | Number of  participants mentioning | Frequency of statements | % of Statements |
| --- | --- | --- | --- | --- | --- | --- |
| Enablers | Reflective motivation | Beliefs about consequences | 1 | 7/8 | 8 | 30% |
|  | Reflective motivation | Identity | 2 | 5/8 | 5 | 19% |
|  | Reflective motivation | Intentions | 3 | 4/8 | 5 | 19% |
|  | Psychological capability | Behaviour Regulation | 4 | 2/8 | 3 | 11% |
|  | Reflective motivation | Optimism | 5 | 2/8 | 2 | 7% |
|  | Social opportunity | Social influence | 5 | 2/8 | 2 | 7% |
| Total | - | - | - | - | 27 | 100.0% |
| Barriers | Social opportunity | Social influence | 1 | 8/8 | 16 | 26% |
|  | Psychological capability | Knowledge / Cognitive and interpersonal (CI) skills | 2 | 8/8 | 14 | 23% |
|  | Psychological capability | Memory, attention and decision making | 3 | 7/8 | 10 | 16% |
|  | Reflective motivation | Beliefs about capabilities | 4 | 6/8 | 8 | 13% |
|  | Physical opportunity | Environmental context and resources | 5 | 5/8 | 6 | 10% |
|  | Automatic motivation | Emotions | 6 | 3/8 | 6 | 10% |
|  | Reflective motivation | Beliefs about consequences | 8 | 1/8 | 1 | 2% |
| Total | - | - | - | - | 61 | 100.0% |

**Table S3.** Main themes in the top ranked enablers and barriers for recently diagnosed T2D patients, with example quotes – before using the GRO Health app.

| Enablers or Barriers | TDF Domain | Themes | Example Quote |
| --- | --- | --- | --- |
| Enablers | Beliefs about consequences | Recognizing the impact of diabetes on dietary choices | Participant 2*: “Yeah, it was the diabetes that sort of made me think, oh, I don't want this, I don't want to be ruled the rest of my life by what I'm going to eat. Well, you have to learn how to manage it, because I think once you're diabetic, the doctor says you're diabetic for life."* |
|  | Identity | Identifying oneself as a diabetic | Participant 7: *"But when I received the diagnosis I thought, ok this is real, I'm ill, I have to start changing things. I just didn’t know where to start actually."* |
|  | Intentions | Intention to adopt a healthier diet | Participant 4: *“And it's in my hands now. And so just doing that it's a case of right, I’m going to educate myself and get a bit healthier.”* |
| Barriers | Social Influence | Lack of professional support/guidance | Participant 8: *“I called my surgery to ask if there was any program to help me and they made me wait half an hour on the phone just to say no program was available for now. And then I asked for another option, but they just said that there was none. So, then I went back searching the internet. Is terrible how they treat you, you know? You want to get better, and you have no support, GP whatsoever.”* |
|  | Knowledge / Cognitive and Interpersonal Skills | Lack of awareness about what represents a correct diet | Participant 4: *“What is the right low carb diet? What percentage of my diet should be proteins, carbs and fats? I had no idea.”* |
|  | Memory, attention and decision making | Difficulty to decide on adequate food and quantities to consume | Participant 3: *“Then you go and search for how much carb is, ok? One place says 50g another says 100g and then you read somewhere else that is better to cut carbs for good. I would look at that and think, ok that’s tricky, I should just pick one and go for it.”* |

**Table S4.** Rank order of enablers and barriers to adopt a heathier diet for longstanding T2D patients – before using the GRO Health app.

| Enablers or Barriers | COM-B | TDF Domain | Rank order | Number of  participants mentioning | Frequency of statements | % of Statements |
| --- | --- | --- | --- | --- | --- | --- |
| Enablers | Social opportunity | Social influences | 1 | 15 | 39 | 25.3% |
|  | Psychological capability | Knowledge | 2 | 14 | 38 | 24.7% |
|  | Psychological capability | Behavioural regulation | 3 | 10 | 28 | 18.2% |
|  | Reflective motivation | Beliefs about capabilities | 4 | 8 | 13 | 8.4% |
|  | Reflective motivation | Beliefs about consequences | 5 | 5 | 10 | 6.5% |
|  | Reflective motivation | Intentions | 6 | 5 | 6 | 3.9% |
|  | Psychological capability | Memory, attention and decision processes | 7 | 4 | 8 | 5.2% |
|  | Reflective motivation | Goals | 8 | 3 | 4 | 2.6% |
|  | Physical opportunity | Environmental context and resources | 9 | 3 | 3 | 1.9% |
|  | Reflective motivation | Social/professional role and identity | 10 | 3 | 3 | 1.9% |
|  | Automatic motivation | Emotions | 10 | 1 | 1 | 0.6% |
|  | Automatic motivation | Reinforcement | 11 | 1 | 1 | 0.6% |
| Total | - | - | - | - | 154 | 100.0% |
| Barriers | Social opportunity | Social influences | 1 | 12 | 31 | 23.7% |
|  | Automatic motivation | Emotions | 2 | 11 | 19 | 14.2% |
|  | Reflective motivation | Beliefs about capabilities | 3 | 10 | 21 | 15.7% |
|  | Psychological capability | Knowledge | 4 | 10 | 18 | 13.4% |
|  | Physical opportunity | Environmental context and resources | 5 | 10 | 17 | 12.7% |
|  | Reflective motivation | Beliefs about consequences | 6 | 8 | 13 | 9.7% |
|  | Physical capability | Physical skills | 7 | 7 | 13 | 9.7% |
|  | Psychological capability | Memory, attention and decision processes | 8 | 1 | 1 | 0.7% |
|  | Reflective motivation | Optimism | 8 | 1 | 1 | 0.7% |
| Total | - | - | - | - | 134 | 100.0% |

**Table S5.** Main themes in the top ranked enablers and barriers for longstanding T2D patients, with example quotes – before using the GRO Health app.

| Enablers or Barriers | TDF Domain | Themes | Example Quote |
| --- | --- | --- | --- |
| Enablers | Knowledge | Knowledge about diabetes and healthy diets | Participant 9: *"I did a lot of research myself and found online a couple of courses that you are supposed to get when newly diagnosed."* |
|  | Environmental context and resources | Availability of professional support/  guidance | Participant 2: *"I was sent on a diabetes training course where basically you learn all about diabetes and what you should not have and what you should have, etc."* |
|  | Behavioural regulation | Actions to self-regulate food intake & lifestyle choices | Participant 6: *“For me it's about, in my head, always calculating how much carbohydrates I've had so far, to determine what I will have."* |
| Barriers | Social influence | Lack of/limited professional support/  guidance | Participant 9: *"* *I was just told to check labels. The diabetic nurse just said anything under 5g of sugar you can take, anything over 5g don’t eat that. That was it. That was the sum and total of the support.”* |
|  | Emotions | Negative emotions experienced upon T2D diagnosis | Participant 13: *"* *When it finally was confirmed, it was a big blow, to be honest with you because I knew I had to do something about it immediately. And you always kind of wanna put things off .”* |
|  | Beliefs about capabilities | Perceived lack of competence to initiate or sustain a healthy diet | Participant 14: *"I also personally don't have the capability for eating meat. That really seems to be required on things like Atkins and keto diets. I eat meat, but I don't eat it in the quantities that they recommend."* |

**Table S6.** Rank order of enablers and barriers to adopt a heathier diet for recently diagnosed T2D patients – using the GRO Health app.

| Enablers or Barriers | COM-B | TDF Domain | Rank order | Number of  participants mentioning | Frequency of statements | % of Statements |
| --- | --- | --- | --- | --- | --- | --- |
| Enablers | Psychological capability | Knowledge | 1 | 6/6 | 31 | 16% |
|  | Psychological capability | Cognitive and interpersonal (CI) skills | 2 | 6/6 | 25 | 13% |
|  | Psychological capability | Behaviour Regulation | 3 | 6/6 | 22 | 11% |
|  | Reflective motivation | Beliefs about capabilities | 4 | 6/6 | 21 | 11% |
|  | Psychological capability | Memory, attention and decision making | 5 | 5/6 | 16 | 8% |
|  | Reflective motivation | Goals | 6 | 5/6 | 16 | 8% |
|  | Reflective motivation | Beliefs about consequences | 7 | 5/6 | 15 | 8% |
|  | Social opportunity | Social influence | 8 | 4/6 | 14 | 7% |
|  | Reflective motivation | Intentions | 9 | 3/6 | 12 | 6% |
|  | Automatic motivation | Emotion | 10 | 2/6 | 8 | 4% |
|  | Physical opportunity | Environmental context and resources | 11 | 3/6 | 7 | 4% |
|  | Automatic motivation | Reinforcement | 12 | 2/6 | 4 | 2% |
|  | Reflective motivation | Identity | 13 | 1/6 | 1 | 1% |
| Total | - | - | - | - | 192 | 100.0% |
| Barriers | Psychological capability | Memory, attention and decision making | 1 | 5/6 | 15 | 34% |
|  | Reflective motivation | Beliefs about capabilities | 2 | 4/6 | 11 | 25% |
|  | Psychological capability | Cognitive and interpersonal (CI) skills | 3 | 3/6 | 8 | 18% |
|  | Social opportunity | Social influence | 4 | 2/6 | 4 | 9% |
|  | Automatic motivation | Emotions | 5 | 2/6 | 3 | 7% |
|  | Reflective motivation | Beliefs about consequences | 5 | 2/6 | 3 | 7% |
| Total | - | - | - | - | 44 | 100.0% |

**Table S7**. Main themes in the top ranked enablers and barriers for recently diagnosed T2D patients, with example quotes – using the GRO Health app.

| Enablers or Barriers | TDF Domain | Themes | Example Quote |
| --- | --- | --- | --- |
| Enablers | Knowledge | Acquired awareness of T2D diets | Participant 3: *"I didn’t know about the low carb diet. I mean, I heard of it before, of course but I didn’t know how to do it. So, I found very helpful to have that in the app. And the way they explain it is very easy to understand, and also, they don’t ask you to stop eating everything so it also made me a lot less paranoid."* |
|  | Cognitive and Interpersonal Skills | Putting the diet into practice | Participant 2: *“my fridge looks much healthier now than they are ever I must admit. And I'm still able to eat a lot. Well, actually, I've not given anything up. It's more a case of the proportions of things that I have a better and the amount of fresh and healthier stuff by now has significantly changed."* |
|  | Behavioural Regulation | Self-monitoring food intake - Log your meals function | Participant 7: *“And then I started like programming myself (through logging). Like if I knew I was going to have pizza at night, I would save my carbs for the night, and eat other things during the day, like a salad for lunch and egg for breakfast."* |
| Barriers | Memory, attention and decision making | Struggle to decide which app features to use | Participant 6: *"It can get quite busy with all the articles and videos… there is no order for you to follow and sometimes I would feel like there is so much going on at the same time here."* |
|  | Beliefs about capabilities | Self-monitoring perceived as too demanding | Participant 4: *"(prompt: have you used the logging features?) Yeah, I have been, I've been doing that I find the monitoring the food is good. But it becomes it's quiet, it's quite difficult when you don't buy stuff that's got a barcode on it. So, when you're mixing lots of things, and then you kind of don't bother putting all the ingredients in because it's a lot of work. So, either you don't do something with too many ingredients or you just don't log it. I decided not to log it."* |
|  | Cognitive and Interpersonal Skills | Lack of/limited cooking skills | Participant 4: *"They suggest a cauliflower rice to replace potatoes, but really if you aren’t a great cook, and I'm not, it’s never going to taste good enough."* |

**Table S8.** Rank order of enablers and barriers to adopt a heathier diet for longstanding T2D patients – using the GRO Health app.

| Enablers or Barriers | COM-B | TDF Domain | Rank order | Number of  participants mentioning | Frequency of statements | % of Statements |
| --- | --- | --- | --- | --- | --- | --- |
| Enablers | Psychological capability | Knowledge | 1 | 15 | 47 | 15.3% |
|  | Psychological capability | Behavioural regulation | 2 | 14 | 56 | 18.2% |
|  | Automatic motivation | Reinforcement | 3 | 13 | 33 | 10.7% |
|  | Reflective motivation | Intentions | 4 | 13 | 29 | 9.4% |
|  | Physical opportunity | Environmental context and resources | 5 | 12 | 53 | 17.3% |
|  | Reflective motivation | Beliefs about consequences | 6 | 11 | 21 | 6.8% |
|  | Psychological capability | Cognitive and interpersonal (CI) skills | 7 | 10 | 30 | 9.8% |
|  | Social opportunity | Social influences | 8 | 10 | 21 | 6.8% |
|  | Reflective motivation | Beliefs about capabilities | 9 | 6 | 11 | 3.6% |
|  | Reflective motivation | Goals | 10 | 3 | 3 | 1.0% |
|  | Automatic motivation | Emotions | 11 | 2 | 2 | 0.7% |
|  | Reflective motivation | Social/professional role and identity | 12 | 1 | 1 | 0.3% |
| Total | - | - | - | - | 307 | 100.0% |
| Barriers | Physical opportunity | Environmental context and resources | 1 | 15 | 84 | 46.9% |
|  | Psychological capability | Memory, attention and decision processes | 2 | 10 | 30 | 16.8% |
|  | Psychological capability | Cognitive and interpersonal (CI) skills | 3 | 9 | 15 | 8.4% |
|  | Social opportunity | Social influences | 4 | 8 | 14 | 7.8% |
|  | Psychological capability | Knowledge | 5 | 6 | 7 | 3.9% |
|  | Reflective motivation | Social/professional role and identity | 5 | 6 | 7 | 3.9% |
|  | Reflective motivation | Beliefs about capabilities | 6 | 5 | 9 | 5.0% |
|  | Reflective motivation | Beliefs about consequences | 7 | 5 | 7 | 3.9% |
|  | Automatic motivation | Emotions | 8 | 4 | 4 | 2.2% |
|  | Physical capability | Physical skills | 9 | 2 | 2 | 1.1% |
| Total | - | - | - | - | 179 | 100.0% |

**Table S9.** Main themes in the top ranked enablers and barriers for longstanding T2D patients, with example quotes – using the GRO Health app.

| Enablers or Barriers | TDF Domain | Themes | Example Quote |
| --- | --- | --- | --- |
| Enablers | Knowledge | Knowledge validation provided by the app | Participant 2: *"There was an awful lot that I found very interesting and that was not necessarily new to me, but it explained why some things are much more important than others, that a lot of the books don't particularly go into."* |
|  | Behavioural regulation | Actions to self-regulate food intake & lifestyle choices | Participant 7: *"And I went back to monitoring what I cooked for a while but altered the proportions and the amounts. And from that I started to realize that carbs were too high and needed to come down."* |
|  | Reinforcement | App elements that incentivize/  motivate participants to adopt/continue with healthy diet | Participant 3: *"And the range of the planning of the weekly plans plus the variety of recipes make it more fun, it's more motivating."* |
| Barriers | Environmental context and resources | Lacking functionalities | Participant 6: *"I couldn't even put most of my medication on there. It just didn't have it. So, it had metformin and that was it."*  Participant 4: *"When I was looking to certain foods and the options that were given, they might need to expand their base."* |
|  | Memory, attention and decision processes | Lack of/limited interest paid to content provided | Participant 11: *"And like I say, I think if I was just starting out with diabetes, I would have engaged with it more. It's because I've got 7 years without… They're trying to compete with what I'm doing already"* |
|  | Cognitive and interpersonal (CI) skills | Limited skills or familiarity engaging with apps | Participant 13: *"For the first two weeks, I didn't know how to access the quizzes, because the quiz button goes to a blank page, but I found out through trial and error.”* |

**Table S10**. Possible intervention strategies for recently diagnosed T2D patients, based on the behavioural diagnosis and APEASE criteria.

| TDF  Domain | Barrier | BCT suggested | Strategy description | APEASE criteria |
| --- | --- | --- | --- | --- |
| Memory, attention and decision making / Skills | Struggle to decide which app features to use | Restructuring the physical environment | Make app interface more user friendly (e.g., simplifying information hierarchy and making it more intuitive) | Affordable, practicable, and acceptable intervention |
|  |  | Instruction on how to perform a behaviour | Deliver an introduction tutorial demonstrating how to use the app’s features in detail | Affordable, practicable, and acceptable intervention |
|  |  | Conserving mental resources | Facilitate content delivery with a more structured interaction and a guided “step-by-step” navigation function | Affordable, practicable, and likely to be accepted by users |
| Cognitive and interpersonal skills | Lack of/limited cooking skills | Feedback on behaviour | Increase visibility and prompts to cook-along videos, when the food logged could be replaced by an existing video with a healthier option | Affordable, practicable, and likely to be accepted by users |
| Beliefs about capabilities | Self-monitoring perceived as too demanding | Demonstration of the behaviour | Provide a small course showing “how to optimize logging and measure ingredients” | Affordable, practicable, and likely to be accepted by users |
|  |  | Feedback on outcomes of behaviour | Prompt further ways to improve logging by offering suggestions after users’ interactions | Affordable, practicable, and likely to be accepted by users |
|  |  | Verbal persuasion about capabilities | Use the credible and friendly tone of voice of the app to provide further video support (e.g, specialized doctor talking about ways to improve patients’ success) | Affordable, practicable, and likely to be accepted by users |

**Table S11**. Possible intervention strategies for longstanding diagnosis T2D patients, based on the behavioural diagnosis and APEASE criteria.

| TDF  Domain | Barrier | BCT suggested | Strategy description | APEASE criteria |
| --- | --- | --- | --- | --- |
| Environmental context and resource | Lacking functionalities | Prompts/cues | Allow users to manually update the frequency and/or timing of their notifications | Affordable and practicable intervention, which is likely to be acceptable and effective |
|  |  | Restructuring the physical environment | Expand the database of products and plates that can be incorporated into the "Log your meal" function | May pose some challenges in terms of practicability and affordability, but likely to be acceptable and effective |
|  |  |  | Ensure technical issues, such as displays taking some time to visualize, are regularly checked and addressed |  |
|  |  | Avoidance/  reducing exposure to cues for the behaviour | Suggest users not to keep unhealthy food at home or to store leftovers right after their meals | Affordable and practicable intervention, likely to be acceptable; effectiveness in terms of impact on the target behaviour would have to be further assessed |
|  |  |  | Suggest users to engage with the app at specific times of the day when they are less busy |  |
| Memory, attention and decision processes | Lack of/limited interest paid to content provided | Prompts/cues | Generate notifications that guide users towards content on trending topics, reflecting new scientific or medical information about nutrition, to increase the likelihood of participants engaging with the app. | This intervention is affordable and practicable, and is likely to be acceptable; effectiveness in terms of impact on the target behaviour would have to be further assessed |
|  |  | Conserving mental resources | Enable pre-populated meal options in the "Food diary", based on previous meal options entered, to reduce memory burden when updating the diary | Affordable and practicable intervention, likely to be acceptable; effectiveness in terms of impact on the target behaviour would have to be further assessed |
|  |  |  | Redesign the landing page of the app to direct the user directly towards the *Getting started* section |  |
| Cognitive and interpersonal skills | Limited skills or familiarity engaging with apps | Instruction on how to perform behaviour | Deliver a tutorial on how to synchronise the GRO Health app with other apps (e.g., blood glucose meter apps, allowing to link blood sugar levels with food intake) | This intervention may be affordable but not practicable, given the number of glucose meters available; acceptability may vary between patients |
|  |  | Graded tasks | Initiate meal plans with the easiest recipes that users can make, and gradually, over time, introduce recipes that may be more complex to prepare | Affordable, practi-cable, and very likely to be acceptable; effectiveness in terms of impact on the target behaviour would have to be further assessed |
