# Supplementary material for: Barriers and Enablers to the Adoption of a Healthier Diet Using an App: Qualitative Interview Study With Patients With Type 2 Diabetes Mellitus
Source: JMIR Diabetes. 2023 Dec 19;8:e49097. doi: 10.2196/49097 (PMC10762608; doi:10.2196/49097)
Supplement: Multimedia Appendix 3 [file diabetes_v8i1e49097_app3.docx]

**Table S12.** BCTs included in the GRO Health App, based on the BCTTv1.

| Grouping | BCT | BCT present? | Instantiation in the GRO Health app |
| --- | --- | --- | --- |
| 1. Goals and planning | 1.1.  Goal setting (behaviour) | ✓ | "Choose your focus" in app set up |
|  | 1.2.  Problem solving | 🗶 | - |
|  | 1.3.  Goal setting (outcome) | ✓ | "Wellness score" calculated on daily basis |
|  | 1.4.  Action planning | ✓ | "Weekly meal planner" |
|  | 1.5.  Review behaviour goal(s) | ✓ | "Log your meal" graphs showing target and actual daily food intake |
|  | 1.6.  Discrepancy between current behaviour and goal | ✓ | "Log your meal" graphs showing target and actual daily food intake |
|  | 1.7.  Review outcome goal(s) | 🗶 | - |
|  | 1.8.  Behavioural contract | 🗶 | - |
|  | 1.9.  Commitment | 🗶 | - |
| 2. Feedback and monitoring | 2.1. Monitoring of behaviour by others without feedback | 🗶 | - |
|  | 2.2. Feedback on behaviour | ✓ | "Log your meal" graphs showing calorie consumption per day |
|  | 2.3. Self-monitoring of behaviour | ✓ | "Log your meal", which serves as a food diary |
|  | 2.4. Self-monitoring of outcome(s) of behaviour | ✓ | "Log your meal" graphs showing calorie consumption per day and over time |
|  | 2.5. Monitoring of outcome(s) of behaviour without feedback | ✓ | "Settings > Apps > Chose services to sync data with" allows to sync data with other apps or devices to track weight, heart rate, blood glucose, physical activity; "Health tracking" function allows to visualize this information |
|  | 2.6. Biofeedback | ✓ | "Settings > Apps > Chose services to sync data with" allows to sync data with other apps or devices to track weight, heart rate, blood glucose, physical activity |
|  | 2.7. Feedback on outcome(s) of behaviour | ✓ | Wellness Score provides feedback on collected data and provides feedback on what collected metrics are, how they can be improved, and provides the tools/resources/activities to do that |
| 3. Social support | 3.1. Social support (unspecified) | ✓ | "Nutrition community > Ask the community", as well as the "Lifestyle > Meet ups" sections allow users to discuss with a moderator and other users on various topics (wellbeing, nutrition, sleep, activity and technical queries) |
|  | 3.2. Social support (practical) | ✓ | "Nutrition community > Ask the community", as well as the "Lifestyle > Meet ups" sections allow users to discuss with a moderator about technical queries regarding the app |
|  | 3.3. Social support (emotional) | 🗶 | - |
| 4. Shaping knowledge | 4.1. Instruction on how to perform the behaviour | ✓ | "Nutrition > Education" videos discuss the target behaviour; the "Getting started section" provides support to engage with the app |
|  | 4.2. Information about Antecedents | ✓ | "Lifestyle > Behaviour Change" videos discuss the antecedents that predict unhealthy eating (e.g., emotional eating) and adopting a healthy diet (e.g., "9 ways to use your lunch break to achieve your health goals") |
|  | 4.3. Re-attribution | 🗶 | - |
|  | 4.4. Behavioural experiments | 🗶 | - |
| 5. Natural consequences | 5.1. Information about health consequences | ✓ | "Nutrition > Education", and "Lifestyle > Expert Opinion" videos discuss health related consequences of adopting a healthy diet |
|  | 5.2. Salience of consequences | 🗶 | - |
|  | 5.3. Information about social and environmental consequences | 🗶 | - |
|  | 5.4. Monitoring of emotional consequences | 🗶 | - |
|  | 5.5. Anticipated regret | 🗶 | - |
|  | 5.6. Information about emotional consequences | 🗶 | - |
| 6. Comparison of behaviour | 6.1. Demonstration of the behaviour | ✓ | "Nutrition > Recipes" show participants how to prepare and cook healthy meals (including list of ingredients, method to prepare meals, pictures of the meal) |
|  | 6.2. Social comparison | 🗶 | - |
|  | 6.3. Information about others’ approval | ✓ | "Nutrition > Recipes" allows for other users to rate the recommended recipes |
| 7. Associations | 7.1. Prompts/cues | ✓ | "Notifications" which serve as prompt/reminders to perform the target behaviour |
|  | 7.2. Cue signalling reward | 🗶 |  |
|  | 7.3. Reduce prompts/cues | ✓ | "Settings > Notifications" allows users to disable notifications or to only enable notification categories of their preference |
|  | 7.4. Remove access to the reward | 🗶 | - |
|  | 7.5. Remove aversive stimulus | 🗶 | - |
|  | 7.6. Satiation | 🗶 | - |
|  | 7.7. Exposure | 🗶 | - |
|  | 7.8. Associative learning | 🗶 | - |
| 8. Repetition and substitution | 8.1. Behavioural practice/rehearsal | 🗶 | - |
|  | 8.2. Behaviour substitution | ✓ | "Nutrition > Lessons > Eating out and takeaways", provides information on how to substitute unhealthy foods and drinks, for healthier options |
|  | 8.3. Habit formation | ✓ | "Notifications" which serve as prompt/reminders to engage with the app, log their meals and perform the target behaviour |
|  | 8.4. Habit reversal | 🗶 | - |
|  | 8.5. Overcorrection | 🗶 | - |
|  | 8.6. Generalisation of target behaviour | ✓ | "Nutrition > Lessons > Eating out and takeaways", provides information on how to eat healthy food when away from home |
|  | 8.7. Graded tasks | 🗶 | - |
| 9. Comparison of outcomes | 9.1. Credible source | ✓ | "Nutrition > Education", and "Lifestyle > Expert Opinion" videos discuss health related topics endorsed by experts |
|  | 9.2. Pros and cons | 🗶 | - |
|  | 9.3. Comparative imagining of future outcomes | 🗶 | - |
| 10. Reward and threat | 10.1. Material incentive (behaviour) | 🗶 | - |
|  | 10.2. Material reward (behaviour) | 🗶 | - |
|  | 10.3. Non-specific reward | 🗶 | - |
|  | 10.4. Social reward | 🗶 | - |
|  | 10.5. Social incentive | 🗶 | - |
|  | 10.6. Non-specific incentive | 🗶 | - |
|  | 10.7. Self-incentive | 🗶 | - |
|  | 10.8. Incentive (outcome) | 🗶 | - |
|  | 10.9. Self-reward | 🗶 | - |
|  | 10.10. Reward (outcome) | 🗶 | - |
|  | 10.11. Future punishment | 🗶 | - |
| 11. Regulation | 11.1. Pharmacological support | ✓ | "Settings > Medications" allows the user to register their medications, encouraging adherence to their treatments |
|  | 11.2. Reduce negative emotions | ✓ | "Lifestyle > Health and wellbeing" offers various resources to reduce negative emotions, such as guided meditations |
|  | 11.3. Conserving mental resources | ✓ | "Nutrition > Recipes or Weekly meal plan" offers lists of ingredients for participants to purchase, reducing the burden on memory when it comes to purchasing healthy food choices |
|  | 11.4. Paradoxical instructions | 🗶 |  |
| 12. Antecedents | 12.1. Restructuring the physical environment | ✓ | "Nutrition > Education and Resources" offers advise on how to change physical environment to adopt a healthy diet (e.g., advise on types of drinks and alcohol to avoid, removing unhealthy food options at home) |
|  | 12.2. Restructuring the social environment | ✓ | "Nutrition > Education and Resources > Behaviour change and a family approach" offers advise on how to change the social environment at home to encourage healthy eating (e.g., eating together, avoid deviating from the shopping list when buying groceries together) |
|  | 12.3. Avoidance/reducing exposure to cues for the behaviour | ✓ | "Nutrition > Education and Resources" provides advise on various topics on how to avoid/reduce exposure to cues for unhealthy eating |
|  | 12.4. Distraction | 🗶 | - |
|  | 12.5. Adding objects to the environment | 🗶 | - |
|  | 12.6. Body changes | 🗶 | - |
| 13. Identity | 13.1. Identification of self as role model | ✓ | "Nutrition > Education and Resources > Behaviour change and a family approach" offers suggests that change implemented by T2D patients can encourage "heathy and long-term behaviour ad skills in younger family members" |
|  | 13.2. Framing/reframing | ✓ | "Nutrition > Education and Resources > Portion control" shifts focus from weighing exact food amounts, to the plate method, a simpler approach to ensure a balanced meal |
|  | 13.3. Incompatible beliefs | 🗶 | - |
|  | 13.4. Valued self-identify | 🗶 | - |
|  | 13.5. Identity associated with changed behaviour | 🗶 | - |
| 14. Scheduled consequences | 14.1. Behaviour cost | 🗶 | - |
|  | 14.2. Punishment | 🗶 | - |
|  | 14.3. Remove reward | 🗶 | - |
|  | 14.4. Reward approximation | 🗶 | - |
|  | 14.5. Rewarding completion | 🗶 | - |
|  | 14.6. Situation-specific reward | 🗶 | - |
|  | 14.7. Reward incompatible behaviour | 🗶 | - |
|  | 14.8. Reward alternative behaviour | 🗶 | - |
|  | 14.9. Reduce reward frequency | 🗶 | - |
|  | 14.10. Remove punishment | 🗶 | - |
| 15. Self-belief | 15.1. Verbal persuasion about capability | ✓ | "Nutrition > Education and Resources" provides content that tells users they can adopt a healthy diet, asserting they can succeed |
|  | 15.2. Mental rehearsal of successful performance | 🗶 | - |
|  | 15.3. Focus on past success | 🗶 | - |
|  | 15.4. Self-talk | 🗶 | - |
| 16. Covert learning | 16.1. Imaginary punishment | 🗶 | - |
|  | 16.2. Imaginary reward | 🗶 | - |
|  | 16.3. Vicarious consequences | 🗶 | - |
| TOTAL (BCTs present) | | **32** | - |

**Table S13**. Select BCTs included in the GRO Health app, compared to the proposed BCTs.

| BCT from BCTTv1 | BCT present? | Instantiation in the  GRO Health app | Proposed BCTs |
| --- | --- | --- | --- |
| *Demonstration of the behaviour* | ✓ | "Nutrition > Recipes" show participants how to prepare and cook healthy meals (including list of ingredients, method to prepare meals, pictures of the meal) | ***Supplements existing BCT*** |
| *Feedback on behaviour* | ✓ | "Log your meal" graphs showing calorie consumption per day | ***Supplements existing BCT*** |
| *Self-monitoring of behaviour* | ✓ | "Log your meal" or food diary | ***Supplements existing BCT*** |
| *Feedback on outcomes of the behaviour* | ✓ | “Wellness Score” provides feedback on collected data and provides feedback on what collected metrics are, how they can be improved, and provides the tools/resources/activities to do that | ***Supplements existing BCT*** |
| *Verbal persuasion about capabilities* | ✓ | "Nutrition > Education and Resources" provides content that tells users they can adopt a healthy diet, asserting they can succeed | ***Supplements existing BCT*** |
| *Instruction on how to perform the behaviour* | ✓ | "Nutrition > Education" videos discuss the target behaviour; the "Getting started section" provides support to engage with the app | ***Supplements existing BCT*** |
| *Prompts/cues* | ✓ | "Notifications" which serve as prompt/reminders to perform the target behaviour | ***Supplements existing BCT*** |
| *Graded tasks* | 🗶 | - | ***New BCT*** |
| *Conserving mental resources* | ✓ | "Nutrition > Recipes or Weekly meal plan" offers lists of ingredients for users to purchase, reducing the burden on memory when buying food | ***Supplements existing BCT*** |
| *Restructuring the physical environment* | ✓ | "Nutrition > Education and Resources" offers advise on how to change physical environment to adopt a healthy diet (e.g., advise on types of drinks to avoid, removing unhealthy food options at home) | ***Supplements existing BCT*** |
| *Avoidance/reducing exposure to cues for the behaviour* | ✓ | "Nutrition > Education and Resources" provides advise on various topics on how to avoid/reduce exposure to cues for unhealthy eating | ***Supplements existing BCT*** |
