# Supplementary material for: Barriers and Enablers to the Adoption of a Healthier Diet Using an App: Qualitative Interview Study With Patients With Type 2 Diabetes Mellitus
Source: JMIR Diabetes. 2023 Dec 19;8:e49097. doi: 10.2196/49097 (PMC10762608; doi:10.2196/49097)
Supplement: Multimedia Appendix 4 [file diabetes_v8i1e49097_app4.docx]

**Discussion.** Constructs used by Nouwen et al [39].

- Autonomous motivation (from Self-Determination Theory - Deci & Ryan, 1985): behaviours that are self-initiated because they are important to the individual and ties into their values and goals system. (i.e., people for whom a healthy diet is part of their value system eat more healthily)
- Self-efficacy (from Social-Cognitive Theory - Bandura, 1986): judgment of one’s abilities to produce given attainments when confronted with impediments and thus determines the amount of effort and perseverance expended in attempting to achieve their goal.
- Self-evaluation: self-monitoring or reflection; assessed as the level of satisfaction/dissatisfaction with one’s level of adherence to recommended dietary self-care activities
- Controlled motivation: controlled behaviours, which occur when a person is pressured either by their interpersonal environment (externally motivated) or intrapsychic forces such as guilt or fear (introjected motivation)
